# Lower Limb Motion Recognition with Improved SVM Based on Surface Electromyography

**DOI:** 10.3390/s24103097

**Published:** 2024-05-13

**Authors:** Pengjia Tu, Junhuai Li, Huaijun Wang

**Affiliations:** 1 College of Computer Science and Technology, Xi’an University of Science and Technology, Xi’an 710054, China; tupengjia@xust.edu.cn; 2 School of Computer Science and Engineering, Xi’an University of Technology, Xi’an 710048, China; wanghuaijun@xaut.edu.cn

**Keywords:** surface electromyography, non-negative matrix factorization, multi-nonlinear features, lower limb motion recognition, GA-PSO-SVM

## Abstract

During robot-assisted rehabilitation, failure to recognize lower limb movement may efficiently limit the development of exoskeleton robots, especially for individuals with knee pathology. A major challenge encountered with surface electromyography (sEMG) signals generated by lower limb movements is variability between subjects, such as motion patterns and muscle structure. To this end, this paper proposes an sEMG-based lower limb motion recognition using an improved support vector machine (SVM). Firstly, non-negative matrix factorization (NMF) is leveraged to analyze muscle synergy for multi-channel sEMG signals. Secondly, the multi-nonlinear sEMG features are extracted, which reflect the complexity of muscle status change during various lower limb movements. The Fisher discriminant function method is utilized to perform feature selection and reduce feature dimension. Then, a hybrid genetic algorithm-particle swarm optimization (GA-PSO) method is leveraged to determine the best parameters for SVM. Finally, the experiments are carried out to distinguish 11 healthy and 11 knee pathological subjects by performing three different lower limb movements. Results demonstrate the effectiveness and feasibility of the proposed approach in three different lower limb movements with an average accuracy of 96.03% in healthy subjects and 93.65% in knee pathological subjects, respectively.

## 1. Introduction

Lower-limb exoskeleton robots have been developed to assist elderly or disabled individuals’ movements in daily activities [1,2,3] and to improve the recovery effect in pathological rehabilitation [4]. The principal to realize the effective control of robotic exoskeletons is accurately inferring the human users’ motion intentions. Among available sensing techniques, surface electromyography (sEMG) signals are preferred for controling information in rehabilitated exoskeleton robots, which mainly consider two factors: sEMG signals could not only detect human user motion intentions ahead of actual physical movements [5], and also directly reflect people’s motion states by muscle contractions. This characteristic of sEMG signal can avoid control delays form exoskeleton robotic applications.

As a bioelectrical signal, sEMG signals contain highly relevant biological information related to human motions. At present, sEMG signals have been extensively performed to recognize human motion intentions in a variety of ways. For example, motion variables (i.e., angle velocity, joint angle, etc.) are continuously estimated. Also, human gait or limb motion modes (i.e., walking, sitting, etc.) are distinguished via machine learning algorithm recognition-based approaches. In particular, many studies have been devoted to recognizing human motion intentions.

In the field of human motion intention recognition, it is crucial to extract features that conform to various motion changes or make motion changes more sensitive. Most existing works aim to directly extract features from sEMG signals, for instance, time domain, frequency domain, time–frequency domain, nonlinear domain, depth features, etc. For instance, Emilia et al. [6] extracted 15 time domain features to recognize lower limb movements, with an average recognition accuracy of over 96.7%. Root mean square (RMS), zero crossing (ZC), slope sign change (SSC), and waveform length (WL) in the time domain, and wavelet transform in the frequency domain, were extracted and fused to achieve over 95% classification accuracy in five lower limb movements [7]. For nonlinear features, Lempel Ziv complexity (LZC) and entropy features were also utilized for motion identification analysis [8,9,10]. Zhang et al. [11] extracted the time domain, frequency domain, and sample entropy (SampEn) and proposed an improved differential evolution (DE) algorithm to optimize the weight value of each feature and achieve the optimal combination of multiple features.

In addition, existing recognition methods of movement classification via machine learning algorithms (i.e., SVM, K-nearest neighbor (KNN), etc.) have also been applied. Since SVM has a superior ability to deal with uncertainties under dynamic conditions, it has been extensively implemented in limb motion classification. For example, a lower limb motion recognition approach based on ReliefF and kernel principal component analysis (KPCA) combined with SVM was proposed. The average recognition accuracy was improved by 10.86% and 2.29% compared with ReliefF-KPCA-BP and ReliefF-KPCA-PNN, respectively [12]. The frequency domain features, such as energy and variance of wavelet packet coefficients were extracted, and a particle swarm optimization–improved SVM classification model was constructed to reach 90.66% classification accuracy in six commonly used upper limb movements [13]. A back propagation (BP) neural network algorithm model based on SVM was proposed, which improved the average recognition accuracy by 9.4% compared with the SVM algorithm in lower limb motions [14]. In particular, SVM parameters have a great influence on the recognition accuracy of movements. Thus, seeking the optimal parameter values for SVM becomes a big challenge in the motion recognition field. Heuristic algorithms (i.e., PSO, whale optimization algorithm (WOA), etc.) are good candidates for optimizing and selecting the SVM model parameters. For instance, an improved WOA algorithm was proposed to reach the optimal parameters of the SVM model [15]. Liu et al. [16] extracted multi-domain features of sEMG signals, and simultaneously established an accurate SVM classification model by proposing an improved WOA. Cao et al. [17] proposed a novel adaptive mutation particle swarm optimization (AMPSO) to optimize the parameters of the SVM algorithm for recognition of sEMG-based limb motions.

Since the muscles associated with lower limb movements overlap each other, the corresponding sEMG signals are complex in nature. Thus, classifying lower limb movements is more challenging for researchers, compared to the recognition of the upper limb movements. However, the objects of most existing methods focus on healthy individuals, with unsatisfactory results for subjects having knee issues. In addition, the functionality of each muscle performing the task-specific lower limb movements (i.e., walking, standing, etc.) is completely different [18]. The knee joint has multiple degrees of freedom movements in flexion, extension, adduction, abduction, medial, and lateral, in which flexion and extension play the most important roles in human lower limb movements and are easy to injure during gait exercise [16,19].

In this paper, the muscles involved in flexion and extension of knee joints are selected to provide the object. A muscle synergy approach is adopted to select the most appropriate muscles. Moreover, nonlinear features of sEMG are believed to be effective in classifying the movements. The Fisher discriminant function method based on the Fisher score (FS) is adopted to reduce the dimension of the multi-nonlinear feature vectors, which makes it conducive for the improved SVM classifier to accuracy recognize lower limb movements. The objective of this paper is to find out the optimal parameter values of the SVM model by proposing an improved hybrid GA-PSO to distinguish various lower limb movements in individuals with and without knee pathology. Hence, an sEMG-based lower limb motion recognition using SVM with an improved hybrid GA-PSO algorithm is proposed. The main contributions are as follows.

- Non-negative matrix factorization (NMF) method is applied to analyze muscle synergy for multi-channel sEMG signal of various lower limb movements so as to select the most appropriate muscles.
- Taking into account the non-linearity and non-stationary of sEMG, we extract the multi-nonlinear features (e.g., approximate entropy (ApEn), SampEn, fuzzy entropy (FuzzyEn), LZC, Lyapunov, and correlation dimension (CD)). Also, the feature selection is performed with the help of the FS based on the Fisher discriminant function method, prior to feeding the dimension-reduced features to the improved SVM.
- Since the hybrid GA-PSO algorithm has both high convergence efficiency and the capability of avoiding being trapped in a local optimal solution, this approach is leveraged to optimize the SVM to find out the best parameters (i.e., penalty factor *p* and kernel function parameters *g*). Simultaneously, a GA-PSO-SVM approach is utilized to construct a lower limb motion recognition model.
- The proposed approach performance has been verified in the task of classifying three lower limb movements associated with knee muscles in healthy individuals (96.03%) and subjects afflicted with knee disorders (93.65%), respectively.

## 2. The Proposed Approach Framework

**Figure 1:**
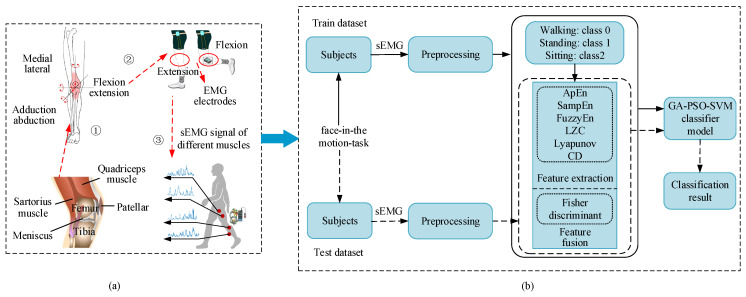
The proposed approach framework. (**a**) Musculoskeletal model of lower limb. (**b**) Lower limb movement recognition model.

sEMG is a kind of bioelectric signal generated with muscle contraction, which drives joint movement and reflects the motion information of the limb. As the electrical signal source of muscle activity, sEMG essentially reflects the movement state of nerve, bone, and muscle systems [16]. Hence, we try to combine the sEMG signal with the motion state of the knee joint musculoskeletal system, as revealed in Figure 1a. Also, the NMF method is utilized to select optimal muscles for various lower limb movements. Specifically, there are two types of subjects with and without knee pathology performing three different lower limb movements (e.g., walking, sitting, and standing). For each subject, in Figure 1b, we label the trials of “walking” as class 0, the trials of “standing” as class 1, and the trials of “sitting” as class 2. We further extract and reduce multi-nonlinear features of sEMG signals as input to an improved GA-PSO-SVM recognition model by classifying three lower limb movements.

### 2.1. Selection of Muscles

Muscle synergy reflects the shared neural drives of motor units across different muscles, which has also been widely applied in limb motion recognition [20]. In this paper, vastus medialis (VM), rectus femoris (RF), biceps femoris (BF), and semitendinosus (ST) are selected as the four most relevant muscles in the lower limb motions, while the contribution of each muscle varies in various movements. Hence, muscle synergy analysis is applied to reduce the data redundancy and obtain the muscles with the greatest contribution during movements. According to muscle synergy theory, the contribution of each muscle in a movement can be obtained through a linear combination of muscle synergies and activation coefficients [16].

(1) $$\begin{array}{c} V_{N\times T}\approx W_{N\times K}\times H_{K\times T}\approx \left[W_{1}W_{2}\cdots ,W_{K}\right]\times \\ {}[H_{1}H_{2}\cdots ,H_{K}{]}^{T}\approx \Sigma_{i=1}^{K}W_{i}H_{i}, \end{array}$$

where *V* is the given muscle activity level matrix, *N* is the channel number, *T* is the time series length, and *K* is the number of muscle synergies. *W* and *H* are the muscle synergy matrix and the activation coefficients matrix. In addition, $W_{i}$ represents the muscle synergy and $H_{i}$ indicates the activation coefficients of the *i* muscle synergy, respectively.

NMF is a decomposition technique, which can transform the sEMG signals from the muscle activity space to the synergy space. More exactly, muscle activation level matrix $V\in R^{m\times n}$ is decomposed into two factors $W\in R^{m\times r}$ and $H\in R^{r\times n}$. The relation is given by,

(2) $$\begin{array}{c} V^{m\times n}=W^{m\times r}\times H^{r\times n}+E^{m\times n}, \end{array}$$

where *m* and *n* are the number of recorded muscles and data samples, *r* indicates the dimension of muscle synergies, and *E* is a matrix of residuals, respectively. In particular, the dimension of muscle synergies is determined based on 90% of the total accumulated data variance. The muscle synergy space $Y\in R^{k\times q}$ is implemented as the input of the muscle synergy-driven musculoskeletal model, where *k* and *q* are mentioned above, respectively.

### 2.2. Multi-Nonlinear Feature Extraction and Selection

#### 2.2.1. Feature Extraction

Different sEMG features can reflect various muscle statuses during movement. Moreover, as sEMG belongs to the nonlinear time series, here, six nonlinear features from sEMG, i.e., ApEn, SampEn, FuzzyEn, LZC, Lyapunov, and CD, are extracted [21]. Specifically, entropy could characterize the complexity of the signal series, and the greater the values are, the more complex the signal is. Also, LZC, Lyapunov, and CD could describe the irregularity, complexity, and dynamical changes of sEMG [10]. Six nonlinear features will be introduced in sequel.

**Entropy**. ApEn relies on the data length [22], and SampEn is an improvement of ApEn, independent of data length and insensitive to missing data [23]. Nevertheless, both ApEn and SampEn measure similarity using a unit step function, with a large mutation and lacking continuity of entropy. Note that FuzzyEn utilizes an exponential function to blur the similarity measure so that entropy changes continuously and smoothly with parameters [24]. In addition, the vector similarity of ApEn and SampEn is determined by the absolute value difference with the data baseline drift. FuzzyEn determines the similarity of vectors by determining the shape of the fuzzy function with an exponential function, thereby blurring the similarity measure. Since the exponential function possesses the two desired properties, continuous ensures the similarity does not change abruptly and convex ensures self-similarity is maximum, the imported exponential functions do not sacrifice precision.

For an *N* sample time series $\left\{x(i):1\leq i\leq N\right\}$, a fixed value of *m* and τ integers and tolerance $r_{1}$, forms vector sequences $\left\{X_{i}^{m},i=1,\cdots ,N-m+1\right\}$ as follows:

(3) $$\begin{array}{c} X_{i}^{m}=\left\{x(i),x(i+1\tau ),\cdots ,x(i+(m-1)\tau )\right\}, \end{array}$$

where $X_{i}^{m}$ represents *m* consecutive *x* values.

The distance $d_{ij}$ between $X_{m}\left(i\right)$ and $X_{m}\left(j\right)$ is as follows:

(4) $$\begin{array}{c} d_{ij}=d\left[X_{i}^{m},X_{j}^{m}\right]=\max_{k\in (0,m-1)}\left\{\left|x(i+k\tau )-x(j+k\tau )\right|\right\}. \end{array}$$

Estimate the probability $C_{i}^{m}\left(r_{1}\right)$ as:

(5) $$\begin{array}{c} C_{i}^{m}\left(r_{1}\right)=\frac{1}{N-(m-1)\tau }\sum_{j=1}^{N-(m-1)\tau }\theta \left(d_{ij}-r_{1}\right) \end{array}$$

and

(6) $$\begin{array}{c} \phi^{m}\left(r_{1}\right)=\frac{1}{N-(m-1)\tau }\sum_{i=1}^{N-(m-1)\tau }\ln C_{i}^{m}\left(r_{1}\right), \end{array}$$

where θ is the Heaviside function,

(7) $$\begin{array}{c} \theta =\left\{\begin{array}{cc} 0, & d_{ij}\leq r_{1}\\ 1, & d_{ij}>r_{1} \end{array}\right.. \end{array}$$

Therefore, for a finite sequence of length *N*, both ApEn and SampEn of the signal are obtained

(8) $$\begin{array}{c} \mathrm{ApEn}\left(m,r_{1},N\right)={Œ}^{m}\left(r_{1}\right)-{Œ}^{m+1}\left(r_{1}\right) \end{array}$$

and

(9) $$\begin{array}{c} \mathrm{SampEn}\left(m,r_{1},N\right)=\ln{Œ}^{m}\left(r_{1}\right)-\ln{Œ}^{m+1}\left(r_{1}\right), \end{array}$$

respectively.

In addition, given *n*, the similarity degree $D_{ij}$ from $X_{i}^{m}$ to $X_{j}^{m}$ is calculated through a fuzzy function $\mu (d_{ij},n,r_{1})$ as:

(10) $$\begin{array}{c} D_{ij}\left(n,r_{1}\right)=\mu \left(d_{ij},n,r_{1}\right)=e^{-{\left(d_{ij}/r_{1}\right)}^{n}}. \end{array}$$

Define the function $\varphi^{m}$ as:

(11) $$\begin{array}{c} \varphi^{m}\left(n,r_{1}\right)=\frac{1}{N-m+1}\sum_{i=1}^{N-m+1}\left(\frac{1}{N-m}\sum_{j=1,j\neq i}^{N-m}D_{ij}\right), \end{array}$$

and thus, the FuzzyEn of the signal is obtained as:

(12) $$\begin{array}{c} \mathrm{FuzzyEn}\left(m,n,r_{1},N\right)=\ln{’}^{m}\left(n,r_{1}\right)-\ln{’}^{m+1}\left(n,r_{1}\right). \end{array}$$

It is remarked that the parameter *m* is defined by the data itself and represents the length of the repeating modes in the sample vectors. Also, the tolerance $r_{1}$ means the constraint on the repeated modes. In general, *m* is set to 2, and $r_{1}$ is 0.15* standard deviation in biological time series analysis [25].

**Lempel-Ziv complexity**. LZC evaluates the chaotic state (that is, complexity) and randomness of the time series signal by measuring the number of different sub-strings and their occurrence rates along a given sequence [9,26].

For a time series $\left\{X=x(1),x(2),\cdots ,x(N)\right\}$ with length *N*, first transform it into a binary symbolic sequence $\left\{S=s(1),s(2),\cdots ,s(N)\right\}$ by,

(13) $$\begin{array}{c} s\left(i\right)=\left\{\begin{array}{cc} 0, & if\;x\left(i\right)<\bar{X}\\ 1, & otherwise \end{array}\right., \end{array}$$

where $\bar{X}$ is the mean value of the signal X(N).

Initialize both S=s(1) and Q=s(2), and set the complexity index c(N)=1. Then, define SQ by combining *S* with *Q*, and $SQ_{r}$ represents the substring obtained from SQ by deleting the last letter of SQ.

Determine whether *Q* belongs to a subsequence of $SQ_{r}$. If so, *Q* is no new pattern at present, the complexity remains unchanged; otherwise, *Q* stands for a new pattern, the complexity index increases. The former processes are represented until all characters in the sequence *S* are traversed to obtain the complexity index c(N).

Note that c(N) indicates signal complexity index and is related to *N*. In general, to avoid the c(N) being affected by the time series length *N*, the normalized LZC complexity index is,

(14) $$\begin{array}{c} \mathrm{LZC}=\frac{c(N)(\log(N)+1)}{N}, \end{array}$$

when *N* is large, LZC can be simplified as:

(15) $$\begin{array}{c} \mathrm{LZC}=\frac{c(N)\log(N)}{N}. \end{array}$$

In particular, the normalized complexity index has upper and lower limits more conducive to the extraction of nonlinear dynamic features.

**Chaotic features**. Motivated by our previous works [21], both CD and Lyapunov are extracted as the chaotic features, to reflect the complexity of sEMG signals.

The phase space *Y* is reconstructed by embedding dimension $m_{1}$ and relay time $\tau_{1}$, i.e.,

(16) $$\begin{array}{c} Y=\left[x_{N},x_{N-\tau_{1}},\cdots ,x_{N-\left(m_{1}+1\right)\tau_{1}}\right]. \end{array}$$

where $m_{1}$ and $\tau_{1}$ are determined by the convergence result and the sampling interval, respectively.

The correlation dimension is expressed by,

(17) $$\begin{array}{c} CD_{m}=\lim_{r\to 0}\frac{\ln CI(r_{2})}{\ln r_{2}}, \end{array}$$

where $CI(r_{2})$ is the association function representing the probability, in which the distance between two points is less than $r_{2}$. Further, both $CD_{m}$ and $CI(r_{2})$ satisfy the logarithmic linear relationship, θ is the Heaviside function,

(18) $$\begin{array}{c} CI\left(r_{2}\right)=\frac{1}{N^{2}}\sum_{j=1}^{N}\sum_{i=1}^{N}\theta (r_{2}-|Y_{i}-Y_{j}\left|\right),i\neq j. \end{array}$$

By the Wolf algorithm [27], the Lyapunov exponent is expressed by,

(19) $$\begin{array}{c} \lambda =\frac{1}{t_{M}-t_{0}}\sum_{k=1}^{M}\log_{2}\frac{L^{\prime }\left(t_{k}\right)}{L(t_{k})}, \end{array}$$

where $L(t_{k})$ is the distance between points $Y(t_{i})$ and its nearest neighbor point, and it is the evolution of the two points with time *t*, *M* is the total number of iterations, respectively.

#### 2.2.2. Features Selection

In the data signal sequel, each feature is denoted by a vector of length 24,

(20) $$\begin{array}{c} f_{i}=[LZC_{VM},LZC_{ST},LZC_{BF},LZC_{RF},CD_{VM},\cdots ,CD_{RF},Ly_{VM},\cdots ,Ly_{RF},\\ SE_{VM},\cdots ,SE_{RF},AE_{VM},\cdots ,AE_{RF},FE_{VM},\cdots ,FE_{RF}]. \end{array}$$

The subscript of a feature vector component stands for the muscle. Hence, a matrix $F\in R^{24\times N_{train}}=\left[f_{1},\cdots ,f_{N_{train}}\right]$ can represent the entire set of features. The Fisher discriminant function, which takes the ratio of inter-class to intra-class dispersion degree as the optimization goal, to maximize the discriminant of various samples [28].

For the training sample $\left\{x(i)\right\}$, $S_{B}$ and $S_{w}$ indicate the inter-class and intra-class spacing matrix, respectively,

(21) $$\begin{array}{c} S_{B}=\sum_{q=1}^{N}N_{q}\left(\bar{x}-{\bar{x}}_{q}\right){\left(\bar{x}-{\bar{x}}_{q}\right)}^{T} \end{array}$$

and

(22) $$\begin{array}{c} S_{w}=\sum_{q=1}^{Q}\sum_{i=1}^{Q_{q}}\left(x_{i}-{\bar{x}}_{q}\right){\left(x_{i}-{\bar{x}}_{q}\right)}^{T}, \end{array}$$

where *Q* and $Q_{q}$ are the number of training sample categories and belonging to class *q*, $\bar{x}$ and $x_{i}$ represent the average vector of all training samples and *i*-th training sample, ${\bar{x}}_{q}$ indicates the average vector of the *q*-th training sample, respectively. Thus, the separability index FS is obtained, i.e., $FS=\frac{\mathrm{tr}(S_{B})}{\mathrm{tr}(S_{w})}$, where, $\mathrm{tr}(S_{B})$ and $\mathrm{tr}(S_{w})$ are traces of the inter-class and intra-class spacing matrix, respectively. Also, the larger the index FS, the more accurate the classification would be achieved in the sample space.

According to FS, that is, $\left\{FS_{1},FS_{2},\cdots ,FS_{6}\right\}$, an optimal set of feature components is selected, which would reduce the computational overheads of subsequent recognition tasks, and hopefully maintain the accuracy of the sEMG classifier.

In short, the Fisher discriminant function maps samples from high-dimensional to low-space, such that the projected samples have large inter-class spacing and small intra-class spacing in low-dimensional space to achieve the optimal separability of samples in low-dimensional space. Thus, an FS is computed for each feature component according to Fisher discriminant function, separately. Next, the top-*q* ranked feature components with large scores are picked to alleviate the complexity and achieve feature dimensionality reduction.

### 2.3. Improved Hybrid GA-PSO Algorithm with SVM

#### 2.3.1. Hybrid GA-PSO Algorithm

PSO, a population-based method, is desirable for regarding each individual in a population as a particle in the search space [29]. Supposing the *D*-dimensional location of the *i*-th particle at iteration time *t* is represented as $L_{i}^{t}=\left\{l_{i1}^{t},l_{i2}^{t},\cdots ,l_{iD}^{t}\right\}$, its velocity is $V_{i}^{t}=\left\{v_{i1}^{t},v_{i2}^{t},\cdots ,v_{iD}^{t}\right\}$, the optimal location found so far (i.e., the personality best position, pbest) is $Lp_{i}^{t}=\left\{lp_{i1}^{t},lp_{i2}^{t},\cdots ,lp_{iD}^{t}\right\}$ until iteration *t*, the optimal location found by the swarm so far (i.e., the global best position, gbest) is $Lg_{i}^{t}=\left\{lg_{i1}^{t},lg_{i2}^{t},\cdots ,lg_{iD}^{t}\right\}$, and then, this particle is updated as follows:

(23) $$\begin{array}{c} v_{ij}^{t+1}=wv_{ij}^{t}+c_{1}r_{3}\left(lp_{ij}^{t}-l_{ij}^{t}\right)+c_{2}r_{4}\left(lg_{ij}^{t}-l_{ij}^{t}\right) \end{array}$$

and

(24) $$\begin{array}{c} l_{ij}^{t+1}=l_{ij}^{t}+v_{ij}^{t+1}, \end{array}$$

where *w* is an inertia weight parameter of the particle and is set as 1, $c_{1}$ and $c_{2}$ indicates the cognition and social learning factor, and $r_{3}$ and $r_{4}$ are random numbers between [0, 1], respectively.

GA [30], an effective optimization algorithm, is desirable to solve various problems in engineering applications. Also, it simulates the process of population evolution and performs a series of genetic operations, such as selection, crossover, and mutation on the current population, to generate a new generation and gradually progress the population to a state close to the optimal solution. Therefore, in this paper, both crossover and mutation in GA are incorporated into PSO to increase the diversity of populations.

Assuming that two individuals $z_{t}^{i},z_{t}^{j}\left(i\neq j\right)$ crossover arithmetically at time  *t*, the two new individuals are generated at time t+1, i.e.,

(25) $$\begin{array}{c} z_{t+1}^{i}=\alpha z_{t}^{j}+\left(1-\alpha \right)z_{t}^{i} \end{array}$$

and

(26) $$\begin{array}{c} z_{t+1}^{j}=\alpha z_{t}^{i}+\left(1-\alpha \right)z_{t}^{j}, \end{array}$$

if α is constant, the crossover operation becomes uniform arithmetic crossover; otherwise, it is non-uniform arithmetic crossover.

The mutation operation has two advantages: it can give GA the ability of local random search and maintain population diversity to prevent premature convergence. For gene operations in the GA, mutation is partially incorporated into PSO to increase the population diversity. In this paper, the position $l_{iD}$ of the *i*-th particle in the *D*-dimensional space in the PSO is replaced by individual $z_{t}^{i}$, and the historical optimal individual $z_{\max}^{i}$ is leveraged to replace the individual optimal $lp_{ij}^{t}$ in PSO. Similarly, the historical optimal species group $z_{\max}^{j}$ is leveraged to replace the global optimal $lg_{ij}^{t}$, and cumulative difference $\Delta z_{\max,t}^{i}$ of $z_{\max}^{i}$ is used to replace $v_{ij}^{t+1}$ as follows:

(27) $$\begin{array}{c} \Delta z_{\max,t}^{i}=\Delta z_{\max,t-1}^{i}+\frac{\left(\Delta z_{\max,t}^{i}-\Delta z_{\max,t-1}^{i}\right)}{t}. \end{array}$$

Substituting (27) into (23) and (24), respectively, the velocity and position update of the mutation are obtained as,

(28) $$\begin{array}{c} \Delta z_{\max,t+1}^{i}=\Delta z_{\max,t}^{i}+c_{1}r_{3}\left(z_{\max}^{i}-l_{ij}^{t}\right)+c_{2}r_{4}\left(z_{\max}^{j}-l_{ij}^{t}\right) \end{array}$$

and

(29) $$\begin{array}{c} l_{ij}^{t+1}=l_{ij}^{t}+\Delta z_{\max,t+1}^{i}. \end{array}$$

To summarize, the hybrid GA-PSO approach can be described in Algorithm 1.
**Algorithm 1** Pseudo-code of GA-PSO.1:Initialize particle swarm size *M*, cognition and social learning factor $c_{1}$ and $c_{2}$ ∈[0,4], random values $r_{3}$ and $r_{4}$ ∈[0,1], maximum iterations *T*, search dimension *N*, particle velocity *V*, particle position *P*, convergence accuracy *C*.2:Conduct selection, crossover and mutation operations for search particle by Formulas (26)–(29).3:Calculate the fitness of each particle. The fitness and its position of the optimal search particle are determined by ranking the fitness.4:**while** t≤T **do**5:    **for** each search particle **do**6:          Update $lp_{ij}^{t}$ and $lg_{ij}^{t}$7:          **if** $lp_{ij}^{t}\leq pbest$ and $lg_{ij}^{t}\leq gbest$ **then**8:             update the velocity and position of search particle by (22) and (23)9:          **end if**10:    **end for**11:    Conduct selection, crossover and mutation operations for the search particle according to Formulas (26)–(29).12:    Calculate the fitness of each search particle. The fitness and its location of the optimal search particle are determined by ranking the fitness.13:    t=t+114:**end while**15:Return the velocity and position of the optimal search particle.

#### 2.3.2. Improved GA-PSO-SVM Algorithm

Admittedly, options abound for the sEMG classification, e.g., LDA, neural network, and SVM. In particular, SVM, is an effective recognition method in lower limb sEMG, but, whose performance would be significantly influenced by the kernel function parameter *g* and penalty factor *p* [31]. Hence, the parameters of SVM should be appropriately set to satisfy a high modeling capability under various identification situations.

The PSO algorithm, as a heuristic algorithm, is a good candidate to optimize and select the model parameters in SVM. However, the PSO is easily trapped in local optimal solutions [29]. The GA algorithm draws on the experience of natural selection and Mendelian laws of heredity to run computational processes with unique chromosome coding and decoding operations. Nevertheless, the coding and decoding process increases the calculation complexity [30]. Also, GA could evaluate multiple solutions in the search space to reduce the risk of falling into a locally optimal solution. Leveraging both GA and PSO, we establish a hybrid algorithm combining both GA and PSO to optimize the SVM model. More specifically, both high convergence efficiency and the ability to avoid falling into local optimal solutions are mainly associated with PSO and GA [32], respectively.

**Figure 2:**
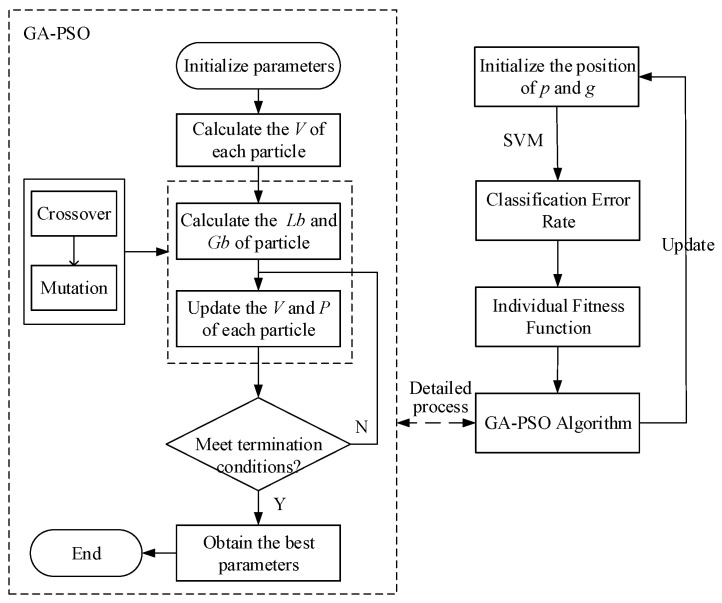
Flow chart of genetic algorithm–particle swarm optimization–support vector machine (GA-PSO-SVM) algorithm for lower limb motion classification.

We aim to optimize the SVM by leveraging a hybrid GA-PSO and applying it to seek the best parameters. In particular, the radial basis function is adopted as a kernel function. Figure 2 describes the chart of the GA-PSO-SVM algorithm for lower limb movement classification, as follows: (i) The parameters *p* and *g* are initialized and search particle $s_{ij}=\left(p_{i},g_{i}\right)$ is set; (ii) SVM classification error rate is defined as the fitness function so that $s_{ij}$ in PSO contains the model parameters to be optimized in SVM; (iii) A hybrid GA-PSO algorithm is utilized to update the individual positions iteratively, and meanwhile, the optimal model parameters $\left(p_{d},g_{d}\right)$ that correspond to the minimum fitness are generated during the optimization process; (iv) GA-PSO-SVM classifier model is constructed to optimize parameters and output the movement classification results.

## 3. Experimental Protocol and Results Discussion

### 3.1. Experimental Protocol

A publicly available dataset is discussed in this study, which can be found in the UCI machine learning repository [33]. This dataset was acquired from 22 male participants older than 18 years of age. Also, this dataset contains signals from eleven subjects with knee normality and abnormality, previously diagnosed by a professional. The sEMG is recorded by the acquisition equipment (MWX8, USA) with a sampling frequency of 1000 Hz, and the real-time data are transmitted through a Bluetooth adapter. Biceps femoris (BF), rectus femoris (RF), semitendinosus (ST), and vastus medialis (VM) are the four relevant muscles to the knee joint flexion and extension movement. In particular, RF and BF are utilized for the extension of the knee joint and flexion of the hip joint, respectively; ST is used for the extension and abduction of the hip joint and flexion of the knee joint; and VM is leveraged for the abduction of the hip joint [34]. Also, each subject performs three different lower limb motions (i.e., walking, standing, and sitting).

### 3.2. Results Analysis and Discussion

#### 3.2.1. Signal Preprocessing

**Figure 3:**
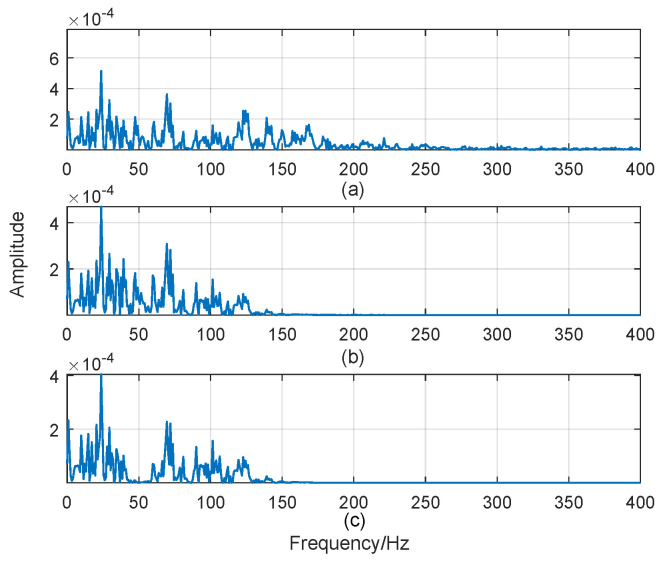
sEMG preprocessing from biceps femoris (BF) muscle during walking movement. (**a**) Raw sEMG spectrum; (**b**) sEMG spectrum from 10 to 150Hz; (**c**) spectrum removing 50 Hz frequency component.

The sEMG is highly sensitive to noises and susceptible to external interference, so preprocessing is required. Since subjects with knee abnormality have slow response, there will be deviations at the beginning of each movement collection. Thus, to avoid this kind of biased data, the 200 ms of front and back segments of each motion data are discarded. In particular, the effective frequency band of the sEMG signal lies between 10 and 400 Hz, and mainly occurs between 10 and 150 Hz [35]. Hence, a fourth order band-pass Butterworth filter with a cut-off frequency of 50 Hz is utilized to effectively reduce the influence of baseline drift and artifact noises. The amplitude of the sEMG signals of four BF muscles corresponding to walking movement with healthy individuals and subjects having knee pathology before and after preprocessing are presented in Figure 3. Results show that the sEMG signal is filtered by both 10–150 Hz band-pass filter and 50 Hz notch filter, to remove high-frequency noises and other artefacts. After filtering, the baseline drift is effectively removed.

#### 3.2.2. Selection of Muscles

**Figure 4:**
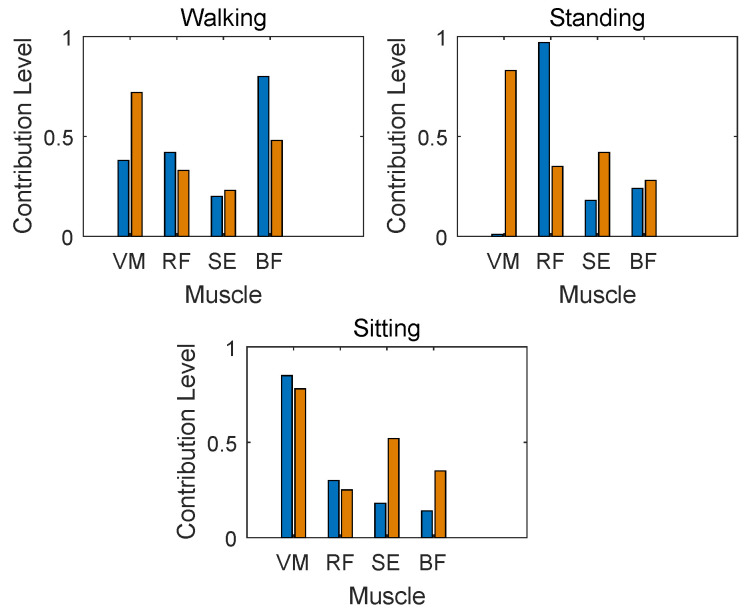
Muscle synergy of various motions (blue and red represent healthy subjects and pathology subjects, respectively).

As can be seen from Figure 4, the contribution level of muscle obtained by the same muscle during various movements and subjects are quite different. Thus, it is necessary to select the muscle with the highest correlation with a specific motion [16]. Nevertheless, how to determine the best muscle for healthy and pathology subjects when performing different lower limb movements is challenging. The four muscles, i.e., RF, BF, VM, and ST, are selected as previously mentioned in Section 3.1. We will derive the contribution level of the selected muscles through muscle synergy. In more detail, the weight coefficient of each muscle is calculated by the NMF method, by analyzing the muscle synergy and obtaining contribution level of all muscles. Muscle synergy of four muscles involved in the three limb movements are presented in Figure 4.

Table 1 reveals the muscle contribution proportions compared to 1 of all subjects about four muscles when performing various three lower limb movements, respectively. From Table 1, we know that the muscle contribution levels of various movements for an individual and various subjects with the same movement are different. For instance, VM and RF own the largest proportion in walking and standing movements while BF accounts for the largest in sitting, respectively. In addition, for healthy subjects, the contribution of VM in walking of Subject 1 is the highest while that of Subject 2 is the lowest. Also, for pathology subjects, the contribution of RF in standing of Subject 4 is the highest while that of Subject 6 is the lowest. This means that the proportion varies significantly between participants. Thus, the muscle selection must be made prior to motion classification. Therefore, VM, RF, and BF are selected here as the muscles for limb recognition during walking, standing, and sitting movements, respectively.

#### 3.2.3. Feature Selection Results

Six nonlinear features (i.e., ApEn, SampEn, FuzzyEn, LZC, Lyapunov, and CD) are extracted from sEMG in two types of subjects (i.e., healthy and pathology subjects) during various lower limb movements. All feature combinations were successively input into the GA-PSO-SVM classification model to recognize lower limb movements. Simultaneously, we leveraged accuracy to evaluate the proposed approach’s performance, as follows, Accuracy=TP+TN/TP+TN+FP+FN, where, TP and FP are true and false positive, TN and FN are true and false negative, respectively.

From Table 2, for healthy subjects, the recognition accuracy based on ${\mathrm{NF}}_{1}$, ${\mathrm{NF}}_{2}$, ${\mathrm{NF}}_{4}$, and ${\mathrm{NF}}_{5}$ is relatively similar, reaching 91.23%, 92.01%, 92.54%, and 90.02%, respectively, while the accuracy of selection feature ${\mathrm{NF}}_{3}$ can reach 97.42%. In addition, for pathology subjects, the recognition accuracy of selection feature ${\mathrm{AF}}_{2}$ (95.38%) is the highest. In total, whether healthy or pathology subjects, with the increase of the dimension of multi-features combination, the recognition accuracy increases. Nevertheless, if all features are directly input into the classification model, it will not only increase the complexity and time of the training model, but also reduce the recognition accuracy of the classifier. In addition, motivated by [21], due to the similarity between the features, there is a situation in which the accuracy decreases with the increase of dimension of multi-feature combination. Hence, it is necessary to discriminate and analyze the FS value of extracted features.

**Figure 5:**
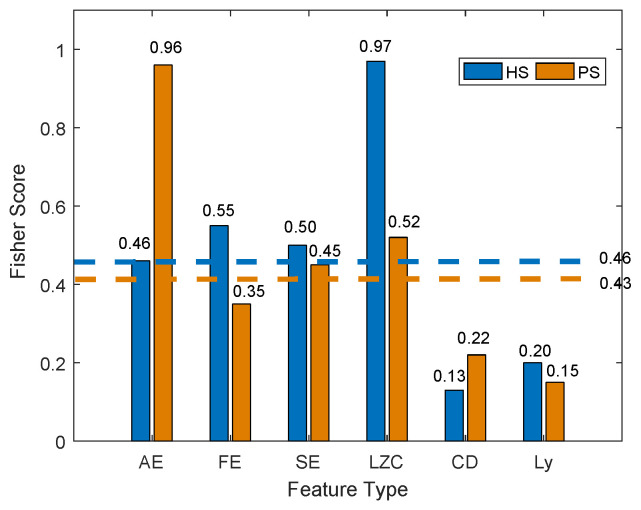
Separability values for six nonlinear features.

It is challenging to distinguish the muscle movement status with all features. Since muscle movement is a gradual process, the change in features before and after muscle movement fatigue is insignificant. Hence, the Fisher discriminant analysis method is utilized to evaluate the score of each feature and find the optimal feature components. Figure 5 demonstrates the average FS value of six features during three lower limb movements, respectively.

From Figure 5, the FS index of each feature finds the difference between either healthy or pathology subjects. That is, according to the FS index, the optimal feature components can be obtained. For a healthy subject, the FS of each feature is ApEn, LZC, SampEn, FuzzyEn, CD, and Lyapunov, in turn. Therefore, we will increase the number of features in the feature combination in the decreasing order of FS index from large to small, that is, ${\mathrm{NF}}_{1}=\left\{\mathrm{LZC},\mathrm{FE}\right\}$, ${\mathrm{NF}}_{2}=\left\{\mathrm{LZC},\mathrm{FE},\mathrm{SE}\right\}$, ${\mathrm{NF}}_{3}=\left\{\mathrm{LZC},\mathrm{FE},\mathrm{SE},\mathrm{AE}\right\}$, ${\mathrm{NF}}_{4}\;=\;\left\{\mathrm{LZC},\mathrm{FE},\mathrm{SE},\mathrm{AE},\mathrm{Ly}\right\}$, and ${\mathrm{NF}}_{5}=\left\{\mathrm{LZC},\mathrm{FE},\mathrm{SE},\mathrm{AE},\mathrm{Ly},\mathrm{CD}\right\}$. Likewise, for pathology subject, the feature combinations are expressed as ${\mathrm{AF}}_{1}=\left\{\mathrm{AE},\mathrm{LZC}\right\}$, ${\mathrm{AF}}_{2}=\left\{\mathrm{AE},\mathrm{LZC},\mathrm{SE}\right\}$, ${\mathrm{AF}}_{3}=\left\{\mathrm{AE},\mathrm{LZC},\mathrm{SE},\mathrm{FE}\right\}$, ${\mathrm{AF}}_{4}\;=\;\left\{\mathrm{AE},\mathrm{LZC},\mathrm{SE},\mathrm{FE},\mathrm{CD}\right\}$, and ${\mathrm{AF}}_{5}=\left\{\mathrm{AE},\mathrm{LZC},\mathrm{SE},\mathrm{FE},\mathrm{CD},\mathrm{Ly}\right\}$, successively. In this paper, the average FS value of the six features is the basis (that is, 0.46 for healthy subject and 0.43 for pathology subject), respectively, the features with FS values higher than average are selected. Consequently, for healthy subject, the optimal feature components are ${\mathrm{NF}}_{1}$, ${\mathrm{NF}}_{2}$, and ${\mathrm{NF}}_{3}$, and ${\mathrm{AF}}_{1}$ and ${\mathrm{AF}}_{2}$ are the optimal feature components of pathology subjects. Thus, ${\mathrm{NF}}_{3}$ and ${\mathrm{AF}}_{2}$ are the optimal feature components for healthy and pathology subjects, respectively.

### 3.3. Experimental Comparison Analysis

#### 3.3.1. Time-Frequency and Nonlinear Feature

To verify the performance of the extracted nonlinear features, we compare them to the time domain and frequency domain features, respectively. Also, the three time-domain features, including RMS, IEMG, and ZC, are extracted, and the frequency domain analysis indicators include MPF and MF. Similarly, these extracted features are also selected.

**Figure 6:**
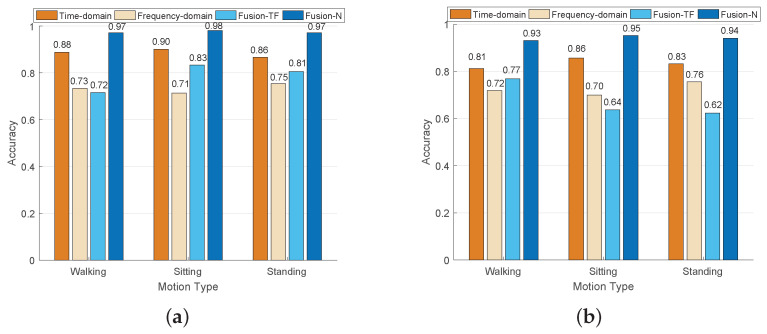
Recognition results of healthy and pathological subjects during three lower limb movements using different features. (**a**) Healthy subjects. (**b**) Pathology subjects.

Figure 6 shows the classification results of four types of movements with multi-domain features (e.g., time domain, frequency domain, selected time–frequency domain, and selected nonlinear domain). In particular, the average recognition accuracy is leveraged to measure the performance of various features and is expressed by Accuracy1+Accuracy2 +⋯+Accuracyn/n; *n* is the number of features. Also, the window size is 300 ms. From Figure 6, the average recognition accuracy based on time domain features, frequency domain features, and selected time–frequency domain features is 88.53%, 73.40%, and 78.67%, respectively, while the average accuracy of the selected nonlinear features can reach 97.40%, for healthy subjects. In terms of the pathology subjects, the average recognition accuracy of extracted nonlinear features (94.10%) is higher than that of the time domain (83.36%), frequency domain (72.67%), and selected time–frequency domain (67.67%), respectively. The results show that the classification accuracy of the selected features in the time–frequency domain is the lowest, and also, the nonlinear features are also higher than that of the time and frequency domain features. The extracted nonlinear features can better reflect the natural attributes of sEMG, rather than random direct extraction of feature parameters.

#### 3.3.2. Different Classifier Algorithms

In addition, to verify the classification performance of the proposed GA-PSO-SVM approach, we compare it with the SVM with gray wolf optimization (GWO-SVM), WOA-SVM, GA-SVM, and PSO-SVM through both the recognition accuracy and time computational. In particular, to avoid possible errors caused by the results of one experiment, each classification model is trained four times. From Table 3, the proposed GA-PSO-SVM method possesses the highest classification accuracy for three lower limb movements, as compared to other optimized SVM classification models. Average recognition accuracy based on WOA-SVM and GA-SVM is relatively similar, reaching 85.33% and 87.16%, respectively, while the accuracy of the proposed method can reach 95.76%. In particular, the average accuracy of GWO-SVM and PSO-SVM is 81.79% and 92.00%, respectively. In terms of the training time of the classification model, the averaged training time of the proposed approach is 16.63 s, which is significantly shorter than other classification models. Experiment results show that the proposed approach could not only improve the recognition accuracy, but also shorten the training time.

## 4. Conclusions

In this paper, a lower limb motion recognition model is constructed by combining the musculoskeletal model and the fused nonlinear chaotic features of sEMG signals. According to the skeletal and muscle motion mechanism during human movements, the musculoskeletal model of lower limb movements is built. In general, sEMG is considered a kind of nonlinear and non-stationary signal with chaotic components. The six nonlinear chaotic features are extracted, and fisher discriminant analysis is utilized for feature fusion. Compared with single features, it has a stronger characterization ability and achieves better results in training and testing models. To solve the problem that SVM is very sensitive to parameter setting, which leads to lower recognition accuracy, a recognition model based on the hybrid GA-PSO algorithm is proposed to optimize the parameters of SVM. The effectiveness of the proposed approach is validated by trials on eleven healthy subjects and eleven knee abnormal subjects, and results strongly support the superior performance of the proposed approach.

With the popularity of sEMG wearable sensing equipment, the lower limb motion recognition has attracted much attention owing to it helping improve human–machine interaction performance in exoskeleton rehabilitation. However, this paper mainly focuses on three lower limb motions. Hence, future works will include researching the practicability of the proposed approach of multi-channel sEMG with complex movements (i.e., crossing obstacles or going up stairs), to enhance the practicability of the proposed approach. Moreover, the reviewer’s valuable suggestions have motivated us to extend the proposed approach to the exoskeleton rehabilitation training model.

## Figures and Tables

**Table 1:** Muscle contributing level about four muscles.

Motions	Muscles	Healthy Subjects			Pathology Subjects		
		Sub.1	Sub.2	Sub.3	Sub.4	Sub.5	Sub.6
Walking	RF	0.2504	0.1368	0.2177	0.0347	0.1213	0.1124
	BF	0.2607	0.1812	0.2314	0.1283	0.1451	0.1722
	VM	0.9019	0.8201	0.8326	0.9081	0.8737	0.8102
	ST	0.2223	0.1740	0.1439	0.1209	0.9383	0.1961
Standing	RF	0.9225	0.8219	0.8452	0.9305	0.9051	0.7086
	BF	0.1480	0.1500	0.1394	0.0941	0.0931	0.1871
	VM	0.0939	0.1240	0.1843	0.1713	0.0875	0.1315
	ST	0.2229	0.1521	0.1568	0.0974	0.1527	0.1852
Sitting	RF	0.1168	0.1254	0.2341	0.1531	0.0928	0.1697
	BF	0.8481	0.7526	0.7246	0.9441	0.9150	0.8875
	VM	0.0909	0.1805	0.1841	0.1895	0.1880	0.1829
	ST	0.1849	0.1860	0.1876	0.1876	0.1950	0.1826

**Table 2:** The recognition accuracy of feature selection (%).

Types		Walking	Standing	Sitting	Average
HS	$NF_{1}$	88.99	93.38	91.30	91.23
	$NF_{2}$	94.38	91.09	90.57	92.01
	$NF_{3}$	97.08	98.05	97.14	97.42
	$NF_{4}$	91.10	94.42	92.09	92.54
	$NF_{5}$	90.75	87.79	91.53	90.02
PS	$AF_{1}$	86.53	83.99	84.95	85.16
	$AF_{2}$	95.13	95.78	95.25	95.38
	$AF_{3}$	92.43	92.20	90.88	91.79
	$AF_{4}$	90.92	91.49	90.01	90.84
	$AF_{5}$	87.93	86.80	87.25	87.32

**Table 3:** Comparison of healthy and pathology subjects with different algorithms (%).

		GWO-SVM	WOA-SVM	PSO-SVM	GA-SVM	Ours
Walking	HS	88.17	90.09	92.37	90.73	97.08
	PS	76.79	86.26	88.99	87.86	93.10
Standing	HS	83.50	83.79	92.75	82.37	97.14
	PS	80.49	84.64	91.10	89.78	94.00
Sitting	HS	83.34	85.46	94.38	90.78	98.05
	PS	78.45	81.69	92.43	91.43	95.20
Average		81.79	85.33	92.00	87.16	95.76
Training time(s)		17.63	16.96	16.85	1 6.75	16.63

## Data Availability

Data are contained within the article.
