# Correction: Tu et al. Lower Limb Motion Recognition with Improved SVM Based on Surface Electromyography. *Sensors* 2024, *24*, 3097

**DOI:** 10.3390/s26133996

**Published:** 2026-06-24

**Authors:** Pengjia Tu, Junhuai Li, Huaijun Wang

**Affiliations:** 1 College of Computer Science and Technology, Xi’an University of Science and Technology, Xi’an 710054, China; tupengjia@xust.edu.cn; 2 School of Computer Science and Engineering, Xi’an University of Technology, Xi’an 710048, China; wanghuaijun@xaut.edu.cn

**Figure/Table Legend**

In the original publication [1], there was a mistake in the legend for Figures 3, 4 and 6 and Table 3. The action labels and group labels were incorrect. Specifically, the group label “walking” was updated to “S-walking”, and also, the group labels “healthy and pathology subjects” were updated to “male and female subjects”, respectively. The correct legend appears below.

**Figure 3**. sEMG preprocessing from biceps femoris (BF) muscle during S-walking movement. (**a**) Raw sEMG spectrum; (**b**) sEMG spectrum from 10 to 150 Hz; (**c**) spectrum removing 50 Hz frequency component.

**Figure 4.** Muscle synergy of various motions (blue and red represent male and female subjects, respectively).

**Figure 6**. Recognition results of male and female subjects during three lower limb movements using different features. (**a**) Male subjects. (**b**) Female subjects.

**Table 3**. Comparison of male and female subjects with different algorithms (%).

**Error in Figure/Table**

In the original publication [1], there was a mistake in Figures 1, 4 and 6 as published. The action labels were incorrect. Specifically, the action labels “Walking”, “Standing” and “Sitting” were updated to “S-walking”, “Q-walking” and “Running”, respectively. 

There was a mistake in Figure 5 as published. The group labels were incorrect. Specifically, the group labels “HS” and “PS” were updated to “male” and “female”, respectively.

The corrected Figure 1, Figure 4, Figure 5 and Figure 6 appear below.

In the original publication [1], there was a mistake in Tables 1–3 as published. The action labels and group labels were incorrect. Specifically, the action labels “Walking”, “Standing” and “Sitting” were updated to “S-walking”, “Q-walking” and “Running”, and also, the group labels “healthy”/“HS” and “pathology”/“PS” were updated to “male” and “female”, respectively. In Table 2, the feature naming was incorrect as well. Specifically, the feature naming “*NF*_1_–*NF*_5_” and “*AF*_1_–*AF*_5_” were updated to “*MF*_1_–*MF*_5_” and “*FF*_1_–*FF*_5_”. In Table 3, the “91.43” was incorrect due to inadvertence. More importantly, the average value of “87.16” also indicates that the “91.43” was incorrect. Specifically, the “91.43” was updated to “81.42”. The corrected Table 1, Table 2 and Table 3 appear below.

**Text Correction**

There was an error in the original publication [1]. The group labels were incorrect. Specifically, the group labels “11 healthy subjects” and “11 knee pathological subjects” were updated to “12 male subjects” and “12 female subjects”, respectively.

A correction has been made to the Abstract:

During robot-assisted rehabilitation, failure to recognize lower limb movement may efficiently limit the development of exoskeleton robots. A major challenge encountered with surface electromyography (sEMG) signals generated by lower limb movements is variability between subjects, such as motion patterns and muscle structure. To this end, this paper proposes an sEMG-based lower limb motion recognition using an improved support vector machine (SVM). First, non-negative matrix factorization (NMF) is leveraged to analyze muscle synergy for multi-channel sEMG signals. Second, the multi-nonlinear sEMG features are extracted, which reflect the complexity of muscle status change during various lower limb movements. The Fisher discriminant function method is utilized to perform feature selection and reduce feature dimension. Then, a hybrid genetic algorithm particle swarm optimization (GA-PSO) method is leveraged to determine the best parameters for SVM. Finally, the experiments are carried out to distinguish 12 male and 12 female subjects by per forming three different lower limb movements. The results demonstrate the effectiveness and feasibility of the proposed approach in three different lower limb movements with an average accuracy of 96.03% in male subjects and 93.65% in female subjects, respectively.

There was an error in the original publication [1]. The description of the individuals was updated. Specifically, “healthy” was updated to “diverse”, and “having knee issues” was updated to “of different genders”. And also, “individuals with and without knee pathology” was updated to “ in various individuals”, respectively.

A correction has been made to Section 1. Introduction, Paragraph 5 and 6:

Since the muscles associated with lower limb movements overlap each other, the corresponding sEMG signals are complex in nature. Thus, classifying lower limb movements is more challenging for researchers, compared to the recognition of the upper limb movements. However, the objects of most existing methods focus on diverse individuals, with unsatisfactory results for subjects of different genders. In addition, the functionality of each muscle performing the task-specific lower limb movements (i.e., walking, standing, etc.) is completely different [18]. The knee joint has multiple degrees of freedom movements in flexion, extension, adduction, abduction, medial, and lateral, in which flexion and extension play the most important roles in human lower limb movements and are easy to injure during gait exercise [16,19].

In this paper, the muscles involved in flexion and extension of knee joints are selected to provide the object. A muscle synergy approach is adopted to select the most appropriate muscles. Moreover, nonlinear features of sEMG are believed to be effective in classifying the movements. The Fisher discriminant function method based on the Fisher score (FS) is adopted to reduce the dimension of the multi-nonlinear feature vectors, which makes it conducive for the improved SVM classifier to accuracy recognize lower limb movements. The objective of this paper is to find out the optimal parameter values of the SVM model by proposing an improved hybrid GA-PSO to distinguish various lower limb movements in various individuals. Hence, an sEMG-based lower limb motion recognition using SVM with an improved hybrid GA-PSO algorithm is proposed. The main contributions are as follows.

There was an error in the original publication [1]. The group labels were incorrect. Specifically, the group labels “healthy individuals” and “subjects afflicted with knee disorders” were updated to “male subjects” and “female subjects”, respectively.

A correction has been made to Section 1. Introduction, the last sentence of the list format of Paragraph 6:

The proposed approach performance has been verified in the task of classifying three lower limb movements associated with knee muscles in male (96.03%) and female subjects (93.65%), respectively.

There was an error in the original publication [1]. The action labels and group labels were incorrect. Specifically, the action labels “Walking”, “Standing” and “Sitting” were updated to “S-walking”, “Q-walking” and “Running”, and also, the group labels “with knee pathology” and “without knee pathology” were updated to “male” and “female”, respectively.

A correction has been made to Section 2. The Proposed Approach Framework, Paragraph 1:

sEMG is a kind of bioelectric signal generated with muscle contraction, which drives joint movement and reflects the motion information of the limb. As the electrical signal source of muscle activity, sEMG essentially reflects the movement state of nerve, bone and muscle systems [16]. Hence, we try to combine the sEMG signal with the motion state of the knee joint musculoskeletal system, as revealed in Figure 1a. Also, the NMF method is utilized to select optimal muscles for various lower limb movements. Specifically, there are two types of subjects, male and female, performing three different lower limb movements (e.g., S-walking, Q-walking and Running). For each subject, in Figure 1b, we label the trials of “S-walking” as class 0, the trials of “Q-walking” as class 1 and the trials of “Running” as class 2. We further extract and reduce multi-nonlinear features of sEMG signals as input to an improved GA-PSO-SVM recognition model by classifying three lower limb movements.

There was an error in the original publication [1]. The description of the UCI dataset was updated to a self-collected dataset, and also, the action labels “Walking”, “Standing” and “Sitting” were updated to “S-walking”, “Q-walking” and “Running”. The completion time of each movement is added at the last sentence. Also, the abbreviation for semitendinosus (ST) was updated to ST.

A correction has been made to Section 3.1. Experimental Protocol, Paragraph 1:

A self-collected dataset is discussed in this study [33], and also, this dataset was acquired from 12 male and 12 female participants older than 20 years of age. The sEMG signal is recorded by the acquisition equipment (i.e., sEMG sensors) with a sampling frequency of 2000 Hz, and the real-time data are transmitted through a Bluetooth adapter. Biceps femoris (BF), rectus femoris (RF), semitendinosus (SE) and vastus medialis (VM) are the four relevant muscles to the knee joint flexion and extension movement. In particular, RF and BF are utilized for the extension of the knee joint and flexion of the hip joint, respectively; SE is used for the extension and abduction of the hip joint and flexion of the knee joint; and VM is leveraged for the abduction of the hip joint [34]. Also, each subject performs three different lower limb motions (i.e., S-walking, Q-walking and Running), and the completion time of each movement is 3 min.

There was an error in the original publication [1]. The group labels and action labels, and their corresponding descriptions were incorrect. Specifically, the action label “Walking” was updated to “S-walking”, and, the group label “healthy” was updated to “male”, respectively. Also, “with knee abnormality” was updated to “could”, and “and subjects having knee pathology” was removed, respectively.

A correction has been made to Section 3.2.1. Signal Preprocessing, Paragraph 1:

The sEMG is highly sensitive to noises and susceptible to external interference, so preprocessing is required. Since subjects could have a slow response, there will be deviations at the beginning of each movement collection. Thus, to avoid this kind of biased data, the 200 ms of front and back segments of each motion data are discarded. In particular, the effective frequency band of the sEMG signal lies between 10 and 400 Hz and mainly occurs between 10 and 150 Hz [33]. Hence, a fourth order band-pass Butterworth filter with a cut-off frequency of 50 Hz is utilized to effectively reduce the influence of baseline drift and artifact noises. The amplitudes of the sEMG signals of four BF muscles corresponding to S-walking movement with male individuals before and after preprocessing are presented in Figure 3. Results show that the sEMG signal is filtered by both the 10–150 Hz band-pass filter and 50 Hz notch filter, to remove high-frequency noises and other artefacts. After filtering, the baseline drift is effectively removed.

There was an error in the original publication [1]. The group labels and action labels were incorrect. Specifically, the action labels “Walking”, “Standing” and “Sitting” were updated to “S-walking”, “Q-walking” and “Running”, and also, the group labels “healthy” and “pathology” were updated to male and female, and the “ST” was updated to “SE”, respectively.

A correction has been made to Section 3.2.2. Selection of Muscles, Paragraph 1 and 2:

As can be seen from Figure 4, the contribution levels of muscle obtained by the same muscle during various movements and subjects are quite different. Thus, it is necessary to select the muscle with the highest correlation with a specific motion [16]. Nevertheless, how to determine the best muscle for male and female subjects when performing different lower limb movements is challenging. The four muscles, i.e., RF, BF, VM and SE, are selected as previously mentioned in Section 3.1. We will derive the contribution level of the selected muscles through muscle synergy. In more detail, the weight coefficient of each muscle is calculated by the NMF method and by analyzing the muscle synergy and obtaining the contribution level of all muscles. The muscle synergies of four muscles involved in the three limb movements are presented in Figure 4.

Table 1 reveals the muscle contribution proportions compared to one of all subjects about four muscles when performing three various lower limb movements, respectively. From Table 1, we know that the muscle contribution levels of various movements for an individual and various subjects with the same movement are different. For instance, VM and RF own the largest proportion in S-walking and Q-walking movements, while BF accounts for the largest in Running, respectively. In addition, for male subjects, the contribution of VM in S-walking of Subject 1 is the highest, while that of Subject 2 is the lowest. Also, for female subjects, the contribution of RF in standing of Subject 4 is the highest, while that of Subject 6 is the lowest. This means that the proportion varies significantly between participants. Thus, the muscle selection must be made prior to motion classification. Therefore, VM, RF and BF are selected here as the muscles for limb recognition during S-walking, Q-walking and Running movements, respectively.

There was an error in the original publication [1]. The group labels and feature naming were incorrect. Specifically, the group labels “healthy” and “pathology” were updated to “male” and “female”, and also, the feature naming “NF_1_–NF_5_” and the feature naming “AF_1_–AF_5_” were updated to MF_1_–MF_5_ and FF_1_–FF_5_.

A correction has been made to Section 3.2.3. Feature Selection Results, Paragraph 1, 2 and 4:

Six nonlinear features (i.e., ApEn, SampEn, FuzzyEn, LZC, Lyapunov and CD) are extracted from sEMG in two types of subjects (i.e., male and female subjects) during various lower limb movements. All feature combinations are successively input into the GA-PSO-SVM classification model to recognize lower limb movements. Simultaneously, we leverage accuracy to evaluate the proposed approach’s performance, as follows, *Accuracy* = *TP* + *TN*/*TP* + *TN* + *FP* + *FN*, where, *TP* and *FP* are true and false positives and *TN* and *FN* are true and false negatives, respectively.

From Table 2, for male subjects, the recognition accuracy based on MF_1_, MF_2_, MF_4_ and MF_5_ is relatively similar, reaching 91.23%, 92.01%, 92.54% and 90.02%, respectively, while the accuracy of selection feature MF_3_ can reach 97.42%. In addition, for female subjects, the recognition accuracy of selection feature FF_2_ (95.38%) is the highest. In total, whether male or female subjects, with the increase in the dimension of multi-features combination, the recognition accuracy increases. Nevertheless, if all features are directly input into the classification model, it will not only increase the complexity and time of the training model but also reduce the recognition accuracy of the classifier. In addition, motivated by [21], due to the similarity between the features, there is a situation in which the accuracy decreases with the increase in the dimension of multi-feature combination. Hence, it is necessary to discriminate and analyze the FS value of extracted features.

From Figure 5, the FS index of each feature finds the difference between either male or female subjects. That is, according to the FS index, the optimal feature components can be obtained. For a male subject, the FS of each feature is ApEn, LZC, SampEn, FuzzyEn, CD and Lyapunov, in turn. Therefore, we will increase the number of features in the feature combination in the decreasing order of the FS index from large to small, that is, MF_1_ = {LZC, FE}, MF_2_ = {LZC, FE, SE}, MF_3_ = {LZC, FE, SE, AE}, MF_4_ = {LZC, FE, SE, AE, Ly} and MF_5_ = {LZC, FE, SE, AE, Ly, CD}. Likewise, for female subject, the feature combinations are expressed as FF_1_ = {AE, LZC}, FF_2_ = {AE, LZC, SE}, FF_3_ = {AE, LZC, SE, FE}, FF_4_ = {AE, LZC, SE, FE, CD} and FF_5_ = {AE, LZC, SE, FE, CD, Ly}, successively. In this paper, the average FS value of the six features is the basis (that is, 0.46 for male subjects and 0.43 for female subjects), respectively; the features with FS values higher than average are selected. Consequently, for male subjects, the optimal feature components are MF_1_, MF_2_ and MF_3_, and FF_1_ and FF_2_ are the optimal feature components of female subjects. Thus, MF_3_ and FF_2_ are the optimal feature components for male and female subjects, respectively.

There was an error in the original publication [1]. The group labels were incorrect. The group labels “healthy subjects” and “pathology subjects” were updated to “male subjects” and “female subjects”.

A correction has been made to Section 3.3.1. Time-Frequency and Nonlinear Feature, Paragraph 2:

Figure 6 shows the classification results of four types of movements with multi-domain features (e.g., time domain, frequency domain, selected time–frequency domain and selected nonlinear domain). In particular, the average recognition accuracy is leveraged to measure the performance of various features and is expressed by *Accuracy1* +⋯+ *Accuracyn/n*, where *n* is the number of features. Also, the window size is 300 ms. As shown in Figure 6, the average recognition accuracy based on time domain features, frequency domain features and selected time—frequency domain features is 88.53%, 73.40% and 78.67%, respectively, while the average accuracy of the selected nonlinear features can reach 97.40% for male subjects. In terms of the female subjects, the average recognition accuracy of extracted nonlinear features (94.10%) is higher than that of the time domain (83.36%), frequency domain (72.67%) and selected time-frequency domain (67.67%), respectively. The results show that the classification accuracy of the selected features in the time–frequency domain is the lowest, and also, the nonlinear features are also higher than that of the time and frequency domain features. The extracted nonlinear features can better reflect the natural attributes of sEMG, rather than the random direct extraction of feature parameters.

There was an error in the original publication [1]. The group labels were incorrect. The group labels “eleven healthy subjects” and “eleven knee abnormal subjects” were updated to twelve male and female subjects.

A correction has been made to Section 4. Conclusions, Paragraph 1:

In this paper, a lower limb motion recognition model is constructed by combining the musculoskeletal model and the fused nonlinear chaotic features of sEMG signals. According to the skeletal and muscle motion mechanism during human movements, the musculoskeletal model of lower limb movements is built. In general, sEMG is considered a kind of nonlinear and non-stationary signal with chaotic components. The six nonlinear chaotic features are extracted, and fisher discriminant analysis is utilized for feature fusion. Compared with single features, it has a stronger characterization ability and achieves better results in training and testing models. To solve the problem that SVM is very sensitive to parameter setting, which leads to lower recognition accuracy, a recognition model based on the hybrid GA-PSO algorithm is proposed to optimize the parameters of SVM. The effectiveness of the proposed approach is validated by trials on twelve male and female subjects, and the results strongly support the superior performance of the proposed approach.

**References Correction**

This correction removes reference entry [33] in the original published version. Citations for former ref. 33 are switched to ref. 35. With this correction, the order of some references has been adjusted accordingly.

The authors state that the scientific conclusions are unaffected. This correction was approved by the Academic Editor. The original publication has also been updated.

## Figures and Tables

**Figure 1:**
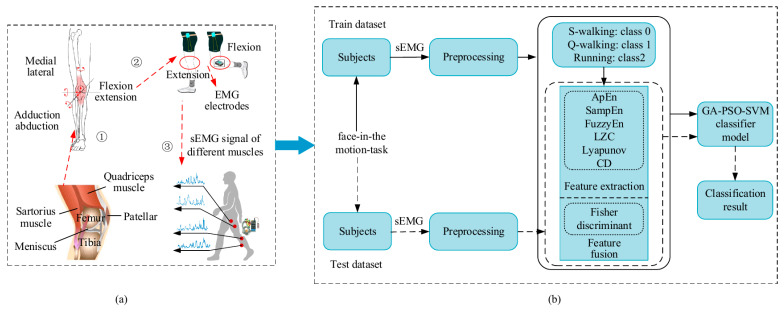
The proposed approach framework. (**a**) Musculoskeletal model of lower limb. (**b**) Lower limb movement recognition model.

**Figure 4:**
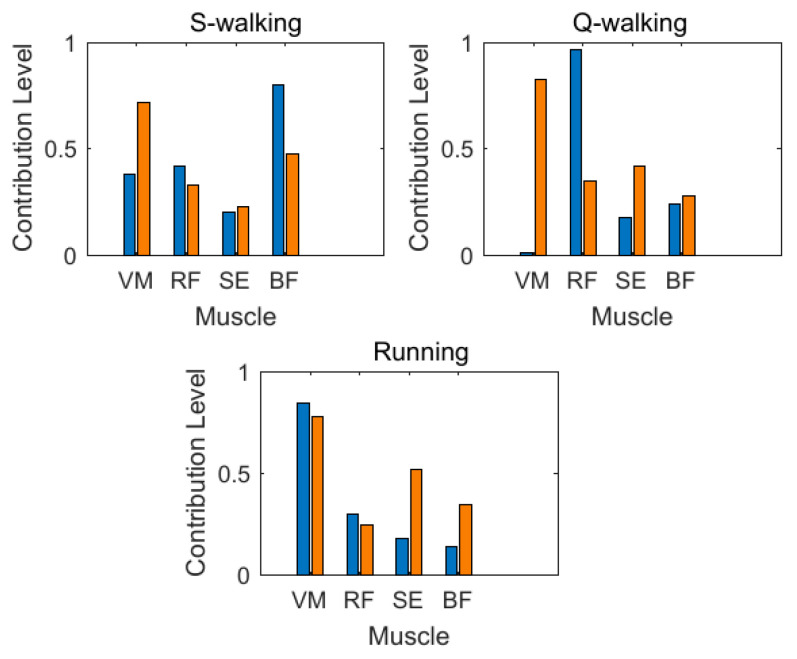
Muscle synergy of various motions (blue and red represent male and female subjects, respectively).

**Figure 5:**
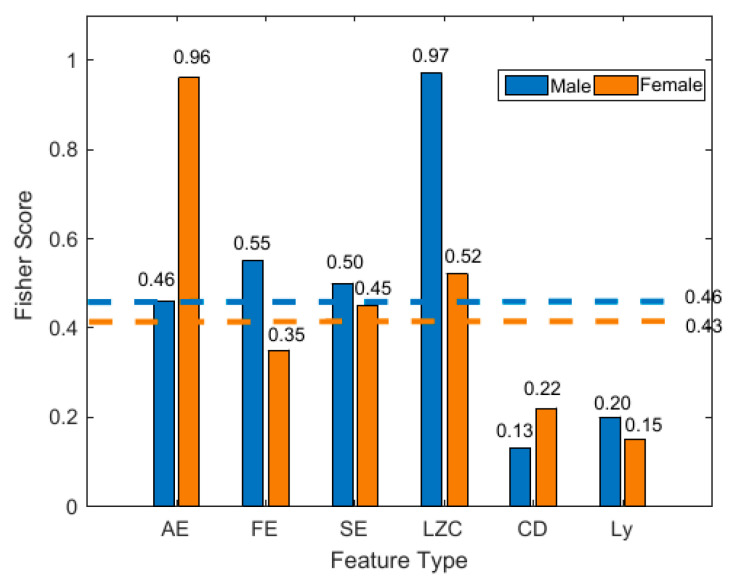
Separability values for six nonlinear features.

**Figure 6:**
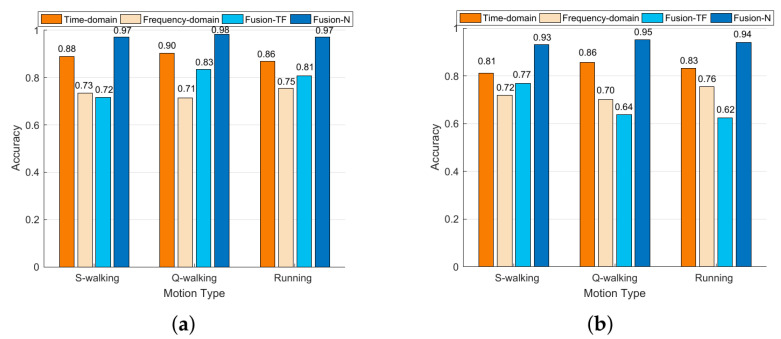
Recognition results of male and female subjects during three lower limb movements using different features. (**a**) Male subjects. (**b**) Female subjects.

**Table 1:** Muscle contributing level about four muscles.

Motions	Muscles	Male Subjects			Female Subjects		
		Sub.1	Sub.2	Sub.3	Sub.4	Sub.5	Sub.6
S-walking	RF	0.2504	0.1368	0.2177	0.0347	0.1213	0.1124
	BF	0.2607	0.1812	0.2314	0.1283	0.1451	0.1722
	VM	0.9019	0.8201	0.8326	0.9081	0.8737	0.8102
	ST	0.2223	0.1740	0.1439	0.1209	0.9383	0.1961
Q-walking	RF	0.9225	0.8219	0.8452	0.9305	0.9051	0.7086
	BF	0.1480	0.1500	0.1394	0.0941	0.0931	0.1871
	VM	0.0939	0.1240	0.1843	0.1713	0.0875	0.1315
	ST	0.2229	0.1521	0.1568	0.0974	0.1527	0.1852
Running	RF	0.1168	0.1254	0.2341	0.1531	0.0928	0.1697
	BF	0.8481	0.7526	0.7246	0.9441	0.9150	0.8875
	VM	0.0909	0.1805	0.1841	0.1895	0.1880	0.1829
	ST	0.1849	0.1860	0.1876	0.1876	0.1950	0.1826

**Table 2:** The recognition accuracy of feature selection (%).

Types		S-Walking	Q-Walking	Running	Average
Male	*MF*_1_	88.99	93.38	91.30	91.23
	*MF*_2_	94.38	91.09	90.57	92.01
	*MF*_3_	97.08	98.05	97.14	97.42
	*MF*_4_	91.10	94.42	92.09	92.54
	*MF*_5_	90.75	87.79	91.53	90.02
Female	*FF*_1_	86.53	83.99	84.95	85.16
	*FF*_2_	95.13	95.78	95.25	95.38
	*FF*_3_	92.43	92.20	90.88	91.79
	*FF*_4_	90.92	91.49	90.01	90.84
	*FF*_5_	87.93	86.80	87.25	87.32

**Table 3:** Comparison of male and female subjects with different algorithms (%).

		GWO-SVM	WOA-SVM	PSO-SVM	GA-SVM	Ours
S-walking	male	88.17	90.09	92.37	90.73	97.08
	female	76.79	86.26	88.99	87.86	93.10
Q-walking	male	83.50	83.79	92.75	82.37	97.14
	female	80.49	84.64	91.10	89.78	94.00
Running	male	83.34	85.46	94.38	90.78	98.05
	female	78.45	81.69	92.43	81.42	95.20
Average		81.79	85.33	92.00	87.16	95.76
Training time (s)		17.63	16.96	16.85	16.75	16.63

## References

[1] Tu P., Li J., Wang H. (2024). Lower Limb Motion Recognition with Improved SVM Based on Surface Electromyography. Sensors.

